# Methods of sample size calculation in descriptive retrospective burden of illness studies

**DOI:** 10.1186/s12874-018-0657-9

**Published:** 2019-01-09

**Authors:** Karissa M. Johnston, Pardis Lakzadeh, Bonnie M. K. Donato, Shelagh M. Szabo

**Affiliations:** 1 Broadstreet Health Economics and Outcomes Research, 343 Railway St Vancouver BC, Vancouver, V6A 1A4 Canada; 2 Memorial University, St John’s, Newfoundland, Canada; 3 Alexion Pharmaceuticals, New Haven, CT USA

**Keywords:** Pharmacoepidemiology, Epidemiology, Public health

## Abstract

**Background:**

Observational burden of illness studies are used in pharmacoepidemiology to address a variety of objectives, including contextualizing the current treatment setting, identifying important treatment gaps, and providing estimates to parameterize economic models. Methodologies such as retrospective chart review may be utilized in settings for which existing datasets are not available or do not include sufficient clinical detail. While specifying the number of charts to be extracted and/or determining whether the number that can feasibly extracted will be clinically meaningful is an important study design consideration, there is a lack of rigorous methods available for sample size calculation in this setting. The objective of this study was to develop recommended sample size calculations for use in such studies.

**Methods:**

Calculations for identifying the optimal feasible sample size calculations were derived, for studies characterizing treatment patterns and medical costs, based on the ability to comprehensively observe treatments and maximize precision of resulting 95% confidence intervals. For cost outcomes, if the standard deviation is not known, the coefficient of variation cv can be used as an alternative. A case study of a chart review of advanced melanoma (MELODY) was used to characterize plausible values for cv in a real-world example.

**Results:**

Across sample sizes, any treatment given with greater than 1% frequency has a high likelihood of being observed. For a sample of size 200, and a treatment given to 5% of the population, the precision of a 95% confidence interval (CI) is expected to be ±0.03. For cost outcomes, for the median cv value observed in the MELODY study (0.72), a sample size of approximately 200 would be required to generate a 95% CI precise to within ±10% of the mean.

**Conclusion:**

This study presents a formal guidance on sample size calculations for retrospective burden of illness studies. The approach presented here is methodologically rigorous and designed for practical application in real-world retrospective chart review studies.

**Electronic supplementary material:**

The online version of this article (10.1186/s12874-018-0657-9) contains supplementary material, which is available to authorized users.

## Background

Observational burden of illness studies are widely used to characterize treatment patterns, resource utilization, costs, and clinical outcomes associated with a disease. In pharmacoepidemiology, burden of illness studies can contextualize the current treatment setting, identify important treatment gaps and their associated consequences, characterize the potential benefits of a new therapy, and provide estimates to parameterize economic models [1–3].

With the increased availability of “big data”, methodological considerations for observational research often focus on the use of large databases – while challenges remain in this setting, available sample size and power do not tend to be problematic given the large pool of individuals from which to draw [4]. However, such databases are not able to answer all burden of illness research questions, due to insufficient clinical detail being recorded, the rarity of the condition, or an appropriate database not being available for a jurisdiction of interest. In this setting, chart review remains a powerful methodology for accessing comprehensive data directly from de-identified patient charts – allowing for real-world burden of illness parameters to be assessed, for example, for individuals with a particular range of laboratory measures or a specific genetic biomarker.

An important limitation to chart review is feasibility of achieving a sufficiently large sample at a reasonable cost. Depending on the setting, charts may not be centrally linked, requiring engagement of individual clinicians to obtain chart access; and if detailed medical data are required to meet study objectives, data extraction may involve several hours per chart. Thus, in the context of a retrospective chart review, a critical a priori consideration is the number of charts that are required to meaningfully address research questions, and the feasibility of identifying and extracting this number of charts. Conversely, researchers may know at the outset how many charts are available to be extracted, and must consider the expected value of the information, before deciding whether to perform chart extraction.

While methodological guidance for conducting retrospective chart reviews has been previously described, formal guidance on sample size calculations is not available [5, 6]. Commonly-used sample size calculations are typically based on a hypothesis-testing framework. Since the outcomes of burden of illness studies tend to be descriptive rather than inferential, such calculations are not aligned with burden of illness objectives, and have limited relevance in this setting. For a burden of illness study the aim of sample size calculation is to ensure sufficient precision in descriptive outcomes, e.g. characterized by the width of 95% confidence intervals (CIs). Given the absence of validated methods for a priori sample size estimates in the context of retrospective chart review studies, the aim of this article was to develop and present rigorous approaches for sample size calculation using a real-world case study.

## Methods

Sample size formulae are presented for parameters that are of frequent interest in the context of a burden of illness study. This includes summarizing treatment patterns (such as the proportion receiving each treatment, and a comprehensive list of therapies used for a condition, including rare therapies), presence of comorbidities, clinical outcomes such as laboratory measures or calculated disease scores, and resource utilization and cost outcomes (including results stratified by subgroup). In practice, multiple outcomes are often of interest and sample size calculations will generate different projections across outcomes; sample size considerations can be focussed on the outcomes of greatest interest, and/or those that generate the largest sample size requirement. The methods described here are generally applicable to categorical (treatment patterns, comorbidity proportion) and continuous outcomes (costs, continuous clinical measures); treatment patterns and costs, respectively, are used as illustrative examples.

### Sample size calculations for categorical outcomes (e.g. treatment patterns)

When considering treatment distributions in a population, assuming a binomial distribution (*n*, *p*) for receiving a particular treatment, in which the *n* represents the sample size and *p* represents the probability of receiving the treatment, the following are direct results of the binomial distribution:

Expected number of observed patients receiving the treatment is:

1 $$ n\kern0.5em \times \kern0.5em p $$

The width of the 95% CI for estimating *p* is:

2 $$ \pm 1.96\kern0.5em \times \kern0.5em \sqrt{\frac{p\left(1-p\right)}{n}} $$

The probability of not receiving the treatment is (1 − *p*), and therefore the probability of all *n* patients not receiving the treatment is (1 − *p*)^*n*^, such that the probability of observing at least one patient receiving the treatment is:

3 $$ 1-{\left(1-p\right)}^n $$

These formulae can be used to define a sample size that ensures all key treatments will be observed, and that the proportions can be estimated within desired precision. To utilize them to generate sample size requirements, limited a priori data are required;. *n* can be selected to yield acceptable values for both quantities. Because the required sample size *n* increases as *p* moves further from 0.50, *p* can be defined to be the most extreme proportion that would be of interest (e.g. a rare treatment given to 1% of the population). Alternatively, sample size requirements can be determined for a range of values of *p*, or, in situations where the maximum sample size is fixed due to other constraints, the corresponding minimum treatment frequency can be calculated.

### Sample size calculations for continuous outcomes (e.g. costs)

When considering medical costs, assuming a normal distribution for mean costs μ and standard deviation σ, precision associated with a particular sample size can be characterized by the width *W* of the 95% CI:

4 $$ W=1.96\kern0.5em \times \kern0.5em \frac{\sigma }{\sqrt{n}} $$

If an estimate of σ is available, e.g. based on published evidence for another jurisdiction or a similar indication, then the width of the CI can be expressed for the maximum feasible sample size *n*. Alternatively, Eq. (4) can be rearranged so that the required *n* can be calculated to obtain 95% CIs for a desired width ± W:

5 $$ n=\frac{1.96^2\sigma }{{\mathrm{W}}^2} $$

Frequently, estimates of σ aren’t available, making sample size calculations challenging – a challenge common across a variety of contexts when estimating sample size. In the absence of such data, one option is to consider the coefficient of variation c_v_, defined as $\frac{\sigma }{\mu }$. Based on this, σ can be expressed as *c*_*v*_  ×  *μ*, and for assumed values of c_v_ and μ, *n* can be estimated without specific estimates for σ. Replacing σ in Eq. (4), the width of a 95% CI can be expressed as:

6 $$ \pm 1.96\kern0.5em \times \kern0.5em \frac{c_v\times \mu }{\sqrt{n}} $$

Via this formula, required sample size *n* can be calculated for a desired with W based on rearranging Eq. (6). If an estimate of μ is available, *n* can therefore be presented for a desired absolute CI width W Eq. (7). Otherwise, if μ is unknown, the desired width could instead be expressed as a desired percentage of the mean, e.g. it is desired to estimate mean cost with a 95% confidence interval precise to within +/− V% of the mean. This is equivalent to saying that $\mathrm{V}=\frac{\mathrm{W}}{\upmu}$, which can be incorporated into Eq. (7); doing this allows for calculation of n without knowing μ or σ Eq. (8). If V is set equal to 100%, the width of the CI would be equal to μ so that the lower bound of the CI for costs would be 0. Smaller values of V, are associated with narrower CIs, entirely above 0.

Expressed with respect to absolute width W, the required sample size is:

7 $$ {\left(\frac{1.96\times {c}_v\times \mu }{V}\right)}^2 $$

Expressed with respect to width V defined as percentage of the mean, the required sample size is:

8 $$ {\left(\frac{1.96\times {c}_v}{V}\right)}^2 $$

A real-world case study is presented below, to describe the precision achieved for a retrospective chart review of burden of illness including treatment patterns and costs in advanced melanoma (the MELODY study) [7], with a total sample size of 655 patients across the United Kingdom (UK), Italy, and France. Costs were presented both per individual overall, as well as per user of specific utilization categories (e.g. hospitalization costs amongst the subgroup with non-zero hospitalization). The range of c_v_ ratios observed in the MELODY study are presented to provide plausible values for future studies.

A sample size calculator based on the formulas presented is available as supplementary material (see Additional file 1).

## Results

Based on Eqs. (1–3), Table 1 presents calculated relationships between sample sizes and the expected number of cases to be observed, the probability of observing a treatment in practice, and expected precision, for a range of treatment probabilities. Across sample sizes, any treatment given with greater than 1% frequency has a high likelihood of being observed. For a sample of size 200, and a treatment given to 5% of the population, the precision of a 95% CI is expected to be ±0.03; i.e. the expected 95% CI would be (0.02–0.08). Generally, with respect to characterizing treatment patterns, sample sizes above 200 are only required for treatments given to 1% of the population or less, or if particularly narrow precision estimates are needed. The information in Table 1 can be used to identify the optimal sample size based on a treatment pattern-related research question, or, in the case of a fixed sample size, to identify the level of detail that can be described.

**Table 1:** Expected number of observed cases; probability and expected precision of observing a treatment in practice

	Expected number of individuals receiving treatment; (Probability of observing treatment at least once) ± Expected 95% confidence interval width for proportion receiving treatment					
	*n* = 50	*n* = 100	*n* = 200	*n* = 300	*n* = 500	*n* = 1000
*p* = 0.01	1 (0.39); ±0.03	1 (0.63); ±0.02	2 (0.87); ±0.01	3 (0.95); ±0.01	5 (0.99); ±0.01	10 (1.00); ±0.01
*p* = 0.05	3 (0.92) ±0.06	5 (0.99) ±0.04	10 (1.00) ±0.03	15 (1.00) ±0.02	25 (1.00) ±0.02	50 (1.00) ±0.01
*p* = 0.10	5 (0.99) ±0.08	10 (1.00) ±0.06	20 (1.00) ±0.04	30 (1.00) ±0.03	50 (1.00) ±0.03	100 (1.00) ±0.02
*p* = 0.25	13 (1.00) ±0.12	25 (1.00) ±0.08	50 (1.00) ±0.06	75 (1.00) ±0.05	125 (1.00) ±0.04	250 (1.00) ±0.03
*p* = 0.50	25 (1.00) ±0.14	50 (1.00) ±0.10	100 (1.00) ±0.07	150 (1.00) ±0.06	250 (1.00) ±0.04	500 (1.00) ±0.03
*p* = 0.75	28 (1.00) ±0.12	75 (1.00) ±0.08	150 (1.00) ±0.06	225 (1.00) ±0.05	375 (1.00) ±0.04	750 (1.00) ±0.03

For cost estimation, based on Eqs. (7) and (8), assuming that an estimate for the standard deviation is not available, an estimate of c_v_ can instead be used. Observed data from the MELODY study are presented in Table 2 to describe a range of c_v_ observed in practice. Trends in observed c_v_ values included higher values typically observed for hospice and hospital costs relative to outpatient costs, and higher when considering the full population of included patients vs. the subset with non-zero use of a particular category of utilization. Across all categories considered, values for c_v_ ranged from 0.26 to 4.30, with a median value of 0.72. In practice, a range of possible values for can be considered, based on any a priori knowledge regarding heterogeneity in the population with respect to health resource utilization and cost outcomes of interest, e.g. the expected range of disease severity, and the anticipated distribution of costs with respect to routine maintenance and care vs. high cost acute treatment such as inpatient stays.

**Table 2:** Observed values of coefficient of variation c_v_ from the MELODY study

	United Kingdom			Italy			France		
	Mean	SD	C_v_	Mean	SD	C_v_	Mean	SD	C_v_
Costs per person									
Hospitalization	3225	7132	2.21	2486	10,689	4.3	6262	6553	1.05
Hospice	2394	4247	1.77	185	396	2.14	298	511	1.72
Outpatient	587	275	0.47	29	15	0.51	28	31	1.11
Costs per user									
Hospitalization	11,437	13,432	1.17	3306	2209	0.67	11,469	8859	0.77
Hospice	10,363	5103	0.49	185	94	0.51	3429	2079	0.61
Outpatient	782	314	0.4	72	28	0.39	59	15	0.26

**Fig. 1:**
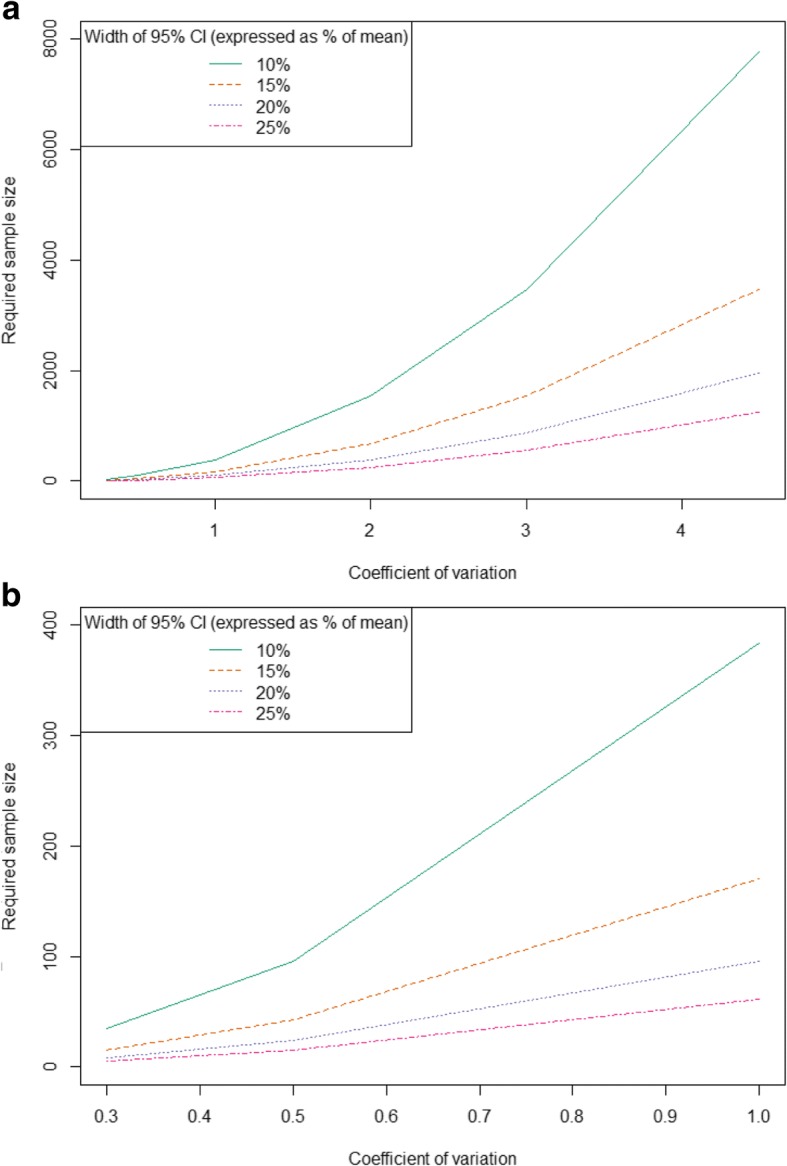
Sample size required across values for coefficient of variation from (**a**) 0–4.5 and (**b**) 0–1.0

Required sample sizes based on Eq. (8) are presented in Fig. 1. Figure 1a displays sample size requirement for the full range of values for c_v_ observed in the MELODY study, while Fig. 1b only considers values for c_v_ between 0 and 1. For the median c_v_ value of 0.72 from the MELODY study, a sample size of approximately 200 would be required to generate a 95% CI precise to within ±10% of the mean. For a c_v_ of 4.5, more than 8000 individuals would be required to estimate a 95% CI precise to within ±10% of the mean. Thus, in situations where large variability is anticipated, e.g. a sample ranging from zero costs to long and costly hospitalizations, the required sample size may be prohibitively large for a chart review or prospective study, requiring access to an administrative or other large database.

When determining optimal sample size, it is important to consider any subgroups of interest, and either target such subgroups directly in sampling strategy or account for expected sample size needs if only the overall population is to be sampled. For example, if descriptive cost analyses are to be conducted for a sample that is estimated to be 20% of the overall population, and a required sample size is identified based on required precision for this subgroup, the full sample will need to be 1/20% = 5x the required sample size identified by the subgroup.

## Discussion

Chart reviews are commonly used to assess clinical outcomes, treatment patterns, and healthcare resource use and costs for more rare health conditions, for very specific indications, or in cases where the required parameters are not captured in large datasets. Despite their ubiquity, methodologic guidance on how to conduct rigorous chart reviews, particularly with respect to selecting appropriate sample sizes, are few. Even the existing methodologic guidance on how to conduct chart reviews provide little direction in this area; suggesting it is beyond the scope of the guidance [6], or assuming a randomized trial-like, hypothesis testing framework [5], rather than methods appropriate for the descriptive objectives that often frame chart reviews. We therefore aimed to fill this gap by providing a framework for estimating appropriate sample sizes for study designs that aim to precisely estimate treatment patterns or resource use parameters, rather than comparing outcomes between groups. These can be especially relevant for situations where the availability of patient charts is limited by time, budget, or the available population size.

We found that for objectives around summarizing categorical and continuous, sample sizes of 100 patients and greater are in most cases sufficient; although larger samples may be required to characterize cost outcomes and/or examine subgroups. For objectives around cost estimation, greater sample sizes are required, particularly if relatively precise estimation is desired, and/or if results specific to particular subgroups are of interest. If 200 to 400 patient charts is the maximum feasible sample size, as is often the case in practice, it can be expected that cost estimate 95% confidence intervals will be precise to within 5–15% of the mean, depending on the ratio of standard deviation to mean costs.

These sample size formulae can be used in two distinct ways. First, if study resources are flexible and desired precision is known, formulae can be used to guide sample size selection. Second, if study resources or available sample size is fixed, formulae can be used to generate anticipated values of precision. We have validated the use of these formulae in a number of other chart review studies [8–10]; and have provided this calculator available online for others to use going forward.

This methodological study addresses an important knowledge gap, as sample sizes are frequently determined using ad-hoc approaches and/or based only on feasibility considerations. Indeed, a non-systematic review of ten recently-published chart reviews focusing on assessing treatment patterns and costs revealed that no studies presented a rationale for their chosen sample size [11–20]. One rule of thumb that has been suggested [6], that is analogous to sample size considerations for regression analyses, is that a minimum of 5–10 charts per variable is required to obtain results that are likely to be both true and clinically useful [21, 22]. However, the number and complexity of outcomes that tend to be considered within a chart review may ultimately limit the utility of this rule in these circumstances, compared to a regression model evaluating the association between a set of independent variables and one dependent variable.

While the methods presented here are broadly applicable to categorical and continuous variables, this does not span the full range of potential outcomes in descriptive burden of illness studies, as resource utilization variables may be analyzed as count data. While a future extension of this work could include formulae based on appropriate distributions (Poisson, negative binomial), the formulas presented for continuous outcomes can be used to generate approximate results based on a normal approximation to the Poisson distribution. For cost data, skewness due to large numbers of zero responses and a few large outlying values may limit the appropriateness of normality assumption; in practice, methods such as two-stage models and/or functional transformations may be undertaken when analyzing data [23]. In sample size estimation, while the assumption of normality for potentially non-normal data is a limitation, using c_v_ values taken from actual cost data will reflect the full scope of variability when such outliers are included and as such are not expected to underestimate required sample size. If doing so yields an infeasibly large sample size, and if statistical techniques are expected to be undertaken at the analysis stage, then this can be incorporated into sample size estimation, e.g. by estimating c_v_ based on a sample with zero-value data points excluded, and/or based on log-transformed data.

The strength of this approach is that it presents a simple, straightforward, validated method for estimating sample size for retrospective studies focusing on multiple descriptive outcomes. In chart reviews, these calculations can be useful during the study design phase, to understand the trade- offs between the expenses in time and money from collecting data from additional charts, versus the additional precision around the estimates that can be obtained. This can be particularly important when considering chart review data as inputs for economic models, where the variability around the estimate can have a major impact. As is the case with estimating sample size a priori for any type of outcome, the applicability of these formulas is limited by the availability of useful preliminary data to use as the basis for the calculations.

A priori estimates of sample size are required when designing chart reviews and other retrospective studies with study objectives that focus on describing treatment patterns, resource use, and costs. However, validated and easily-implemented methods to estimate sample size in this situation are not readily available, or frequently used**.** The approach presented here is methodologically rigorous and designed for practical application in real-world retrospective chart review studies.

## Conclusion

This study presents a formal guidance on sample size calculations for retrospective burden of illness studies. The approach presented here is methodologically rigorous and designed for practical application in real-world retrospective chart review studies and can be used in two distinct ways; where [1] the study resources are flexible and desired precision is known, formulas can be used to guide sample size selection, or [2] if study resources or available sample size is fixed, formulas can be used to generate anticipated values of precision.

## Additional file

Additional file 1: Sample size calculator. The authors have provided an Excel sample size calculator as supplementary material (Additional file 1) to help guide editors and reviewers through the approach presented. If the article is published, the authors are interested in expanding the Excel calculator to an interactive website to facilitate use of the methods by other researchers. (XLSX 15 kb)

## Abbreviations

CI: Confidence Intervals
UK: United Kingdom
